# Classic Kaposi's sarcoma in morocco: clinico -epidemiological study at the national institute of oncology

**DOI:** 10.1186/1471-5945-11-15

**Published:** 2011-11-11

**Authors:** Hassan Errihani, Narjisse Berrada, Soundouss Raissouni, Fadoi Rais, Hind Mrabti, Ghizlane Rais

**Affiliations:** 1 Medical oncology department, National Institute of Oncology, Rabat, Morocco

**Keywords:** CKS, Morocco, clinical features, HHV-8

## Abstract

**Background:**

Classic Kaposi's sarcoma (CKS) is a rare disease likely associated with human herpes virus 8 (HHV-8) infection, and occurs predominantly in Jewish, Mediterranean and middle eastern men .There is a dearth of data in Moroccan patients with CKS regarding epidemiology, clinical characteristics and outcomes. This report examines a cohort of patients with CKS evaluated at the national institute of oncology over 11-year period.

**Methods:**

A retrospective analysis of patients referred to the national institute of oncology with classical Kaposi sarcoma, between January 1998 and February 2008, was performed. Reviewed information included demographics, clinical and pathological staging, death or last follow-up.

**Results:**

During the study period, 56 patients with a diagnosis of CKS have been referred to our hospital. There were 11(19,7%) females and 45 (80,3%) males (male-to-female ratio: 4:1). Mean age at diagnosis was 61,7 ± 15 (range: 15- 86 years). Nodules and/or plaques were the most frequent type of lesion. The most common location was the lower limbs, particularly the distal lower extremity (90%). In addition to skin involvement, visceral spread was evident in 9 cases. The most common visceral involvement sites were lymph nodes (44%), lung (22%), and gastrointestinal tract (22%). Associated lymphoedema was seen in 24 (42%) of the patients. There were 18 stage I patients (32,14%), 8: stage II (14,28%), 21 stage III(37,5%) and 9 stage IV (16,07%). A second primary malignancy was diagnosed in 6 cases (10,7%), none of the reticuloendothelial system.

With a median follow-up of 45 months, 38 (67,8) patients are alive, of whom 25 (65,78%) patients with stable disease, five with progressive disease currently under systemic chemotherapy and 8(21,05%) are alive and free of disease, over a mean interval of 5 years.

**Conclusion:**

This is the largest reported series in our context. In Morocco, CKS exhibits some special characteristics including a disseminated skin disease at diagnosis especially in men, a more common visceral or lymph node involvement and a less frequent association with second malignancies.

## Background

Classic Kaposi's sarcoma (CKS) represents a complex angioproliferative disease characterized by the appearance of spindle-shaped cells. [1].

It was first described by Moritz Kaposi in Vienna in 1872, as a rare and indolent angioproliferative neoplasm, mainly present as a skin lesion [2]. It occurs predominantly in elderly people, particularly men from Eastern Europe and Mediterranean region. The incidence of CKS varies greatly with ethnic and geographic factors. The discovery of Human Herpes virus 8 (HHV-8), also known as Kaposi's sarcoma-associated herpesvirus (KSHV) improved our knowledge of the pathogenesis of Kaposi's sarcoma (KS). HHV-8 can be found in all epidemiological and histological forms of KS [3] and can directly be detected in spindle cells [4].

Tumor lesions are characterized by slow progression, purple macules on the distal portion of the limbs. This lesions could acquire nodular characteristics when the disease progresses. Dissemination to internal organs may occur in more advanced forms.

There is a lack of data in Moroccan patients with CKS regarding clinical features, prognosis, and outcome. The primary aim of this analysis is to outline the clinico-epidemiological profile and outcomes of patients with CKS in the National Institute of Oncology

## Methods

A retrospective analysis of patients referred to the National Institute of Oncology, Rabat, Morocco between January 1998 and February 2008 with a diagnosis of CKS was performed. Patients were identified from the institution tumor registry. KS diagnosis was clinically suspected and histologically confirmed. Serum samples from all patients have been tested for antibodies to HIV, using ELISA and Western Blot. HIV seropositive individuals have been excluded, as well as patients who had received immunosuppressive therapy prior to KS development. Data were collected from the patients' medical files and the following parameters were recorded: gender, origin, age at diagnosis, anatomic distribution (size, location, multicentricity, immunocompromised conditions, second malignancies, clinical course, and date of death or last follow-up if date of death unknown).

The date of diagnosis was defined as the date of biopsy. All patients underwent physical examination, haematological and biochemical analysis, and chest radiography. When clinically indicated, gastrointestinal endoscopy, abdominal ultrasound or computerized tomography (CT) scan were done. HHV-8 antibodies were identified in only 5 patients. The HHV-8 serology was not realized in our institution before 2007. Concerning HHV-8 antibody identification, an immunofluorescence assay was used. This is a commercially available test (Kit Biotrin HHV-8-IgG-IFA), using the indirect immunofluorescent method of antibody detection and titre determination, providing both qualitative and quantitative results to the laboratory. Patient samples are incubated with HHV-8 antigen, which has been stabilised on a glass slide. If HHV-8 IgG antibodies are present in the sample, a stable complex is formed between the antibody and the antigen on the slide. Bound HHV-8 IgG antibody is then reacted with a fluorescein conjugated goat anti-human IgG and this complex is visualised with the aid of a fluorescence microscope.

Unlike AIDS-associated KS, CKS has no universally accepted stage classification. In medical files, stage of the disease were not assessed but can be deducted from the dermatological examination. A recently proposed staging system is often followed, which is based on objective criteria that more closely follow the clinical variability of CKS; to assess the severity of the CKS, the new staging system was used retrospectively, based on objective criteria and the follow up of clinical variability of CKS. It describes four stages (I-IV) based on skin lesions, their localization, the presence or absence of complications and visceral involvement [5].

Follow-up was arranged as a control visit 1 month after the end of treatment, then four visits in a year (each 3 month) for 2 years and then two visits each a year.

### Statistical analysis

SPSS13.0 software was used for statistical analysis. Descriptive of clinical data were expressed in percentage or median or mean ± SD. Age and sex were compared between the two groups by stage (I-II vs III- IV) using a T test for means and χ^2 ^statistic for frequencies.

### Consent and statement of ethical approval

As the treatment of each patient was decided by the medical staff of the centre, oral consent was obtained from the subjects and was approved by the institutional review boards of the National Institute of Oncology, Cancer Centre in Rabat. This study was approved by the institutional review boards of National Institute of Oncology, in Rabat.

## Results

### Patients' characteristics

Fifty six patients were identified to have a diagnosis of CKS between January 1998 and February 2008. A mean of five new patients was diagnosed each year. There were 11(%) females and 43 (%) males (male-to-female ratio: 4:1). Mean age at diagnosis was 61,7 ± 15 (range: 15- 86 years).

The median duration to consultation after the first appearance of lesions was 12 months (range 1-240 months). All patients were North African origin but no ethnic characteristic, especially Jewish, were identified.

Thirteen percent of patients were smokers. Non-insulin dependant diabetes mellitus was found in 2 cases (3,9%). Prostatic adenocarcinoma was diagnosed in 2 patients, a lung cancer in 3 patients and a colic cancer in one patient. No other second primary malignancies especially related to the lymphoreticular system was detected. No familial cases of Kaposi's sarcoma were found.

### Clinical features and location of lesions

The clinical pattern of KS in our patients was characterized by its extreme variability and represented all stages of Kaposi Sarcoma: patch (n = 2; 3,5%), plaque (n =17; 30%) and one with ulceration nodular lesions (n= 21; 37%) and 18 with multinodularity, papulonodular (n = 3; 5%) and maculonodular (n = 13; 23%). The nodular lesions were additionally noted to be ulcerated in 25 cases. Lymphedema was present in 24 patients (42%).

Lower limbs were involved in 50 (89,5%) patients, in 22 of whom the disease was confined to this area. The lesions were developed on both lower and upper extremities in 25(44,6%) subjects. Twelve of these concerned other areas of the body, in particular head and trunk were involved as well. Penis lesions were found in five cases. The lesions were disseminated throughout the body in 8 patients. In addition to skin involvement, visceral spread was evident in 9 cases. The most common visceral involvement sites were lymph nodes (44%), lung (22%), and gastrointestinal tract (22%). HHV-8 serology realized in five patients was positive. Patients' characteristics are summarized in Table 1.

**Table 1:** Patients' characteristics

Characteristics	N(%)
Age at diagnosis	
Mean ± SD	61,7 ± 15,2
Range	15-86

Gender	
Male	45 (80,3%)
Female	11 (19,7%)

Location of lesion	
Skin	56 (100%)
Extracutaneous	9 (16%)

Second primary malignancies	
Present	2 (3,5%)
Absent	54 (96,5%)

Stage at diagnosis	
I	18 (32, 1%)
II	9 (16%)
III	20 (35, 7%)
IV	9 (16, 2%)

Distribution of lesions.	
Lower limbs	50
Both upper and lower extremities	21
Trunk	8
Visceral organs	9
Head and Neck	9
Genital organs	5

Type of lesions	
Patch	2
Plaques	17
Nodules	21
Maculonodular	3
Papulonodular	13

Twenty seven patients were classified in stage I-II (18 in stage I and 9 in stage II) and 29 in stage III- IV (Table 2). Among those in stage I-II, 70% were men, with a mean (SD) age of 60,7 (16,87) years. The corresponding figures for those in stage IV were 96% and 62,7 (14,05) years, respectively. Mean age did not differ significantly by clinical stage (p = 0,59). However, men had significantly more advanced stages than women (P = 0.002) (table 2).

**Table 2:** Staging of the Studied Population

	Staging of disease	
Characteristics	I-II	III-IV

No. (%) of patients	27(48%)	29 (52%)

Sex, No. (%)*		
Male	17 (70%)	28 (96%)
Female	10 (30%)	1 (4%)

Age, y, mean (SD)†	60,7 (16,87)	62,7 (14,05)

*P = .002, stage I-II vs stage III-IV.

†P = .59 stage I-II vs stage II- IV

### Clinical course

Over a follow-up period ranging from 10 to 132 months, with a median of 45 months, 7 (12%) of the CKS patients died of other medical conditions, particularly cardiovascular diseases, two (3,5%) of other malignancies, three (5,3%) of treatment-related complications and six (10,7%) from widespread visceral CKS. Thirty eight patients (67%) are alive, of whom 25 with stable disease, five with progressive disease currently under systemic chemotherapy, and 8 are alive and free of disease, over a mean interval of 5 years.

## Discussion

Kaposi Sarcoma is a solitary or multifocal lymphatic tumor, initially described as Classic Kaposi Sarcoma almost 140 years ago, in elderly men of Middle Eastern and Mediterranean descent [6,7]. No recent report has evaluated the demographics and clinical presentations of CKS patients in Morocco. The current study represents a large retrospective review of 56 patients with a diagnosis of CKS seen at our institution over 11-year period (1998-2008) and is to our knowledge the largest series in our context focusing on this particular population.

Most of demographic and clinical features of our study group are not totally in accordance with previous findings in the literature.

As the analysis of our results indicates, CKS in Morocco exhibits some special clinico-epidemiological characteristics including nodular lesions as first presentation, a more frequent association with lymphedema, disseminated skin lesions at diagnosis, unusual visceral or lymph node involvement and rare coexistence of second primary neoplasm.

The National institute of oncology, Rabat, is the most important cancer center in Morocco, to which are referred patients from the sixteen regions of the country.

In Morocco, we do not possess a national cancer registry, however the local cancer registry of Rabat in 2005, reported a standardized incidence rate of 0,39/100000 habitants. CKS is not uncommon in Morocco, with sporadic cases all over the country. Geographical distribution of CKS according to the region of origin supposes that the disease is more common in northern Morocco. However statistical data regarding the place of birth in general Moroccan population are lacking and no genetic or environnemental factors can be defined.

The incidence of CKS varies greatly with ethnic and geographic factors. The predisposition of Jews has been described in the literature. In Israel, rates of CKS of 2.07 in men and 0.75 in women per 100,000 were calculated [8]. The risk for Jews to develop CKS is 10 times higher than for non-Jews [9]. CKS is rare in North America and North Europe. Low rates were reported in England and Wales as well as in Denmark; intermediate rates were reported in Sweden, whereas higher rates were reported in Italy [10]. The highest incidence rates in Europe were reported in two Mediterranean Italian islands: Sardinia and Sicily. At the National Institute of Oncology, Rabat, CKS accounts 0,1% of all new cases of cancer with a mean of 5 cases diagnosed each year.

The identification of HHV-8 as the causative agent of KS has greatly enhanced our understanding of the pathogenetic events that lead to the development of this tumor [11]. A strong direct correlation between HHV-8 prevalence and CKS incidence was documented. HHV-8 now is known to be the primary cause of all types of KS [12], but CKS occurs in only a small fraction of HHV-8 infected people. In the Mediterranean area, classical KS develops annually in only 0.03% of the HHV-8 infected men older than 50 years and in 0.01%-0.02% of the HHV-8-infected women older than 50 years [12]. Various cofactors have been implicated in the pathogenesis of KS, including genetic susceptibility, immunologic alterations, and endocrine factors [13,14]. It has been hypothesized that the abundant expression of various proinflammatory cytokines in early KS lesions may create an immunologic microenvironment that stimulates the growth of HHV-8 and promotes the development of clinical lesions.[15,16].

One study is available about HHV-8 frequency among CKS patients in Morocco. Kassemi et al reported the seroprevalence of anti-HHV8 antibodies in 2 groups of HIV negative Moroccan patients. Among the 26 patients, 24 (92%) presented with anti-HHV8 antibodies, whereas the 26 donors were seronegative for HHV-8 [17]. In our series, the seroprevalence of anti-HHV8 were not explored. However the serology of anti HHV-8 was done in 5 patients in which it was positive.

The mean age of onset in our series is mainly in accordance to other similar reports [18] . In fact, CKS is a disease of the elderly with a median age at diagnosis reported variously from 50 to 80 years [19,20]. Population-based incidence studies from the United States and Europe reported that the median age at time of diagnosis is the seventh decade of life and only 4-8% of cases were reported in individuals younger than 50 years [10,21]. In the current study, 10 patients were younger than 50 years and the youngest patient was a 15 years old. Worldwide, only sporadic cases occurring before the age of 30 years and occasional cases of CKS have been reported in children [22]. In Israel the median age at onset of CKS is 67 years (range,11-91 years) with no significant variation noted between people from different countries of origin [23,24]. From 1961 until 1989, 13% of the cases of CKS reported in Israel involved people age, 55 years; 0.3% were age, 15 years, with no variation reported during the period.

Interestingly, 90% women in our study were of postmenopausal age (49-84 years), providing additional support to the speculation that female sex hormones may act prophylactically to CKS development [18].

CKS has an overwhelming male predominance, with a male-to-female ratio of approximately 10:1 to 15:1 [10,18]. However, in more recent studies, the sex ratio is much lower: 8:1 in Colombia [25], and 3:1 in Italy [26]. The 4:1 male-to-female ratio found in our study has been reported previously [10,27].

Cutaneous KS is often first evident as discrete erythematous or violaceous bilaterally symmetric patches, most commonly on the lower extremities. A study of Sardinian CKS found this initial lower extremity location evident in 155 of 200 patients [28]. Patches may evolve into plaques and nodules. Nodules and/or plaques were the most common type of lesion in our series.

Associated lymphedema, especially of the feet, is not uncommon and it occurred particularly in a high proportion (42%) of our patients.

KS is a multicentric neoplasm frequently evident as a multiple vascular cutaneous and mucosal nodules [29,30]. Lesions may be limited to skin or sometimes arises to oral cavity, lymph nodes, or viscera in 10-15% [31]. The gastrointestinal tract is the most common extracutaneous site of involvement. An endoscopic upper gastrointestinal tract evaluation of 87 Greek CKS patients showed that 71 (81.6%) had gastrointestinal lesions [32]. Other visceral organs that may be affected include liver, heart and lung [23,33].

Our study reveals more locations in upper extremities, lymph nodes, and visceral sites than previously reported for CKS [33,34]. Unusual sites, as seen in 14 of our patients, include penis, ear, mouth, eyelids, conjunctiva, and nose. They are often associated with poor prognosis [20]. The findings of a large number (n = 5) of CKS in the penis in the current study may relate to an aggressive form of the disease.

Prognosis appears to correlate with the degree of immunosuppression and older age among classic KS patients[35,36]. Localized nodular KS has the best prognosis, with few deaths directly attributable to KS. Clinical classification of KS may be the best prognosticator, comparing localized nodular disease, locally aggressive disease, and generalized KS.

Several staging systems have been proposed for the classification of CKS [37,38]. A recently proposed staging system is often followed, which is based on objective criteria that more closely follow the clinical variability of CKS, and thereby makes therapeutic choices easier. We elected this staging classification because it allows a better distinction between early (skin-localized) and advanced (disseminated) CKS.

CKS by itself is rarely the cause for the patient's demise. In the current study CKS is more aggressive than reported in the literature, with 29 (51,7%) having multicentric disease and classified in stage III and IV and 16% with widespread disseminated disease. Furthermore, the proportion of patients with systemic involvement in our cohort was slightly higher to that seen in other series of patients with CKS, ranging from 4% to 10% of patients.[10,36]. Also, in this work, we found that men were more likely to have advanced stage at diagnosis. Such observations were not previously noted. For example, Stratigos and al reported a series of Sixty-eight patients. Mean age and sex did not differ significantly by clinical stage (I-II vs IV)

It is not a novel observation that CKS is associated with other primary malignancies, often of the reticuloendothelial system, that may precede, coincide with or follow the occurrence of CKS [39]. CKS is reportedly more commonly a primary than a secondary neoplasm [34]. However, information was obtained in several cases indicating CKS arose subsequent to other malignancy, offering the suggestion of a common etiology. The current study found that only 10,7% of CKS patients with complete follow-up data available had associated a second primary malignancy, a lower occurrence than previously reported in the literature, 9-42%.[33,40-42]. This lower frequency of second primary malignancies is similar to the 18% found in a previous study on Israeli patients by Feurman and al [43].

The association between aggressive clinical course of CKS and immunosuppression has previously been suggested. The immunological dysfunction underlying CKS is unknown and may result from advanced age or chronic infection [19]. Its induction by immunosuppressive therapy and its subsequent regression on removal of immunosuppression provided early clinical recognition of the reversibility of CKS. However, none of our patients reported an immunologic dysfunction supporting the previous report of an immunological etiology of CKS [44].

Diabetes mellitus seems to occur more frequently among CKS patients than in the general population [31]. The high frequency of non-insulin dependent diabetes mellitus has been reported repeatedly among American [45] and Israeli [43] subjects but was not found in subjects with endemic form of CKS [46]. The frequency 3,9% of non-insulin dependent diabetes mellitus in our series is lower than reported in the literature.

Only a small number of familial cases of CKS have been reported[35,46,47]. Among Ninety CKS subjects described by Di Giovana [35], only one was familial. Similarly, in a series of 56 CKS patients from north-east Sardinia, no familial cases were reported.[48]

## Conclusion

CKS in Morocco is predominantly a male disease which exhibits some special characteristics, including disseminated skin disease at diagnosis, often accompanied by lymphedema, more common visceral or lymph node involvement and a less frequent association with second malignancies. Although disease may persist, Classic Kaposi Sarcoma by itself is rarely the cause of the patient's death. Extensive epidemiologic studies in Morocco are required.

## Lists of abbreviations

**CKS**: Classic kaposi's sarcoma; **KS: **Kaposi's sarcoma; **HHV-8**: Human Herpes Virus 8; **CT**: computerized tomography;

## Competing interests

The authors declare that they have no competing interests.

## Authors' contributions

RG, BN, RS, FR, EH have conceived the study, exploited data, drafted and wrote the manuscript. RG, EH, HM: corrected and approved the version submitted for publication. All authors read and approved the final manuscript.

## Authors' information

H. E: Professor on medical oncology at the national institute of oncology

R.G: Resident on medical oncology at the national institute of oncology

B.N: Resident on medical oncology at the national institute of oncology

S.R: Resident on medical oncology at the national institute of oncology

H.M: Resident on medical oncology at the national institute of oncology

## Pre-publication history

The pre-publication history for this paper can be accessed here:

http://www.biomedcentral.com/1471-5945/11/15/prepub

## References

[B1] Giuseppe Di Lorenzo Update on classic Kaposi sarcoma therapy New look at an old disease Critical Reviews in Oncology/Hematology 2008 68 242 249 10.1016/j.critrevonc.2008.06.007 18657433

[B2] Schwartz RA Kaposi's sarcoma: an update Journal of Surgical Oncology 2004 87 146 151 10.1002/jso.20090 15334644

[B3] Noel JC Hermans P Andre J Fayt I Simonart T Verhest A Herpesvirus-like DNA sequences and Kaposi sarcoma. Relationship with epidemiology, clinical spectrum, and histologic features Cancer 1996 77 2132 2136 10.1002/(SICI)1097-0142(19960515)77:10<2132::AID-CNCR26>3.0.CO;2-V 8640682

[B4] Boschoff C Schulz TF Kennedy MM Graham AK Fisher C Thomas A Kaposi sarcoma-associated herpesvirus infects endothelial and spindle cells Nat Med 1995 1 1274 1278 10.1038/nm1295-1274 7489408

[B5] Brambilla L Boneschi V Taglioni M Ferrucci S Staging of classic Kaposi's sarcoma: a useful tool for therapeutic choices Eur J Dermatol 2003 13 83 6 12609790

[B6] Kaloterakis A Papasteriades C Filiotou A Hla in familial and nonfamilial Mediterranean Kaposi's sarcoma in Greece Tissue Antigens 1995 45 117 119 10.1111/j.1399-0039.1995.tb02427.x 7792757

[B7] Kaposi M Idiopathisches multiples pigmentsarom der haut Arch f Dermatol u Syph 1872 3 265 273

[B8] Guttman-Yassky E Bar-Chana M Yukelson A Linn S Friedman- Birnbaum R Bergman R Epidemiology of classic Kaposi's sarcoma in the Israeli Jewish population between 1960 and 1998 Br J Cancer 2003 89 1657 60 10.1038/sj.bjc.6601313 14583765 PMC2394430

[B9] Wahman A Melnick SL Rhame FS The epidemiology of classic, African and immunosupressed Kaposi's sarcoma Epidemiol Rev 1991 13 178 199 1765111 10.1093/oxfordjournals.epirev.a036068

[B10] Iscovich J Boffetta P Franceschi S Azizi E Sarid R Classic Kaposi sarcoma: epidemiology and risk factors Cancer 2000 88 500 17 10.1002/(SICI)1097-0142(20000201)88:3<500::AID-CNCR3>3.0.CO;2-9 10649240

[B11] Chang Y Cesarman E Pessin MS Identification of herpes-like DNA sequences in AIDS-associated Kaposi's sarcoma Science 1994 266 1865 1869 10.1126/science.7997879 7997879

[B12] Schwartz Robert A Kaposi sarcoma A continuing conundrumj am acad dermatol 2008 59 2 10.1016/j.jaad.2008.05.001 18638627

[B13] Foster CB Lehrnbecher T Samuels S An IL-16 promoter polymorphism is associated with a lifetime risk of development of Kaposi's sarcoma in men infected with the human immunodeficiency virus Blood 2000 96 2562 2567 11001912

[B14] Cassoni P Sapino A Deaglio S Oxytocin is a growth factor for Kaposi's sarcoma cells: evidence of endocrine-immunological cross talk Cancer Res 2002 62 2406 2413 11956104

[B15] Samaniego F Gallo RC Gupta S Immunopathogenesis of Kaposi's sarcoma Immunology of HIV infection 1996 New York, NY: Plenum Publishing Corp 437 450

[B16] Barillari G Buonaquro L Fiorelli V Effects of cytokines from activated immune cells on vascular cell growth and HIV-1 gene expression: implications for AIDS-Kaposi's sarcoma pathogenesis J Immunol 1992 149 3727 3734 1431144

[B17] El Kassimi B Maladie de Kaposi et anticorps anti-herpès virus-8 au Maroc Médecine et maladies infectieuses 2003 33 226 228 John D

[B18] Stratigos Potouridou Irene Katoulis Alexander C Hatziolou Eftichia Christofidou Eleftheria Stratigos Alexander Hatzakis Angelos Stavrianeas Nicholas G Classic Kaposi's sarcoma in Greece: a clinico-epidemiologicai profile International Journai of Dermatology 1997 36 735 740 10.1046/j.1365-4362.1997.00284.x 9372346

[B19] Einesmith TH Shrum JP Kaposi's sarcoma Int Journal Dermatol 1994 33 755 62 10.1111/j.1365-4362.1994.tb00983.x 7822076

[B20] Safai B Kaposi's sarcoma: a review of the classical and epidemic forms Ann NY Acad Sci 1984 437 378 82 10.1111/j.1749-6632.1984.tb37157.x 6398650

[B21] Di Lorenzo G Kreuter A Di Trolio R Activity and safety of pegylated liposomal doxorubicin as first-line therapy in the treatment of non-visceral classic Kaposi's sarcoma: a multicenter study J Invest Dermatol 2008 128 1578 80 10.1038/sj.jid.5701215 18185536

[B22] Dutz W Stout AP Kaposi's sarcoma in infants and children. [Review on the subject of over 1,200 cases up to 1958] Cancer 1960 13 684 94 10.1002/1097-0142(196007/08)13:4<684::AID-CNCR2820130408>3.0.CO;2-G 13818924

[B23] Iscovich J Boffetta P Brennan P Classic Kaposi's sarcoma in Arabs living in Israel, 1970-1993: a population-based incidence study Int J Cancer 1998 77 319 21 10.1002/(SICI)1097-0215(19980729)77:3<319::AID-IJC1>3.0.CO;2-R 9663588

[B24] Iscovich J Boffetta P Winkelmann R Brennan P Azizi E Classic Kaposi's sarcoma in Jews living in Israel, 1961-1989: a population-based incidence study AIDS 1998 12 2067 72 10.1097/00002030-199815000-00019 9814876

[B25] Garcia A Olivella E Valderrama S Rodriguez G Kaposi's sarcoma in Golombia Cancer 1989 64 2393 8 10.1002/1097-0142(19891201)64:11<2393::AID-CNCR2820641133>3.0.CO;2-9 2804932

[B26] Lospalluti M Mastrolonardo M Loconsole E Glassical Kaposi's sarcoma: a survey of 163 cases observed in Bari, south Italy Dermatology 1995 191 104 8 10.1159/000246525 8520054

[B27] Wahman A Melnick SL Rhame FS The epidemiology of classic, African and immunosuppressed Kaposi's sarcoma Epidemiol Rev 1991 13 178 199 1765111 10.1093/oxfordjournals.epirev.a036068

[B28] Montesu M Rossella M Cottoni F Le sedi nel sarcoma di Kaposi classico. Studio su una casistica di 200 pazienti [in Italian] G Ital Dermatol Venereol 1998 133 247 50

[B29] Schwartz RA Kaposi's sarcoma: advances and perspectives J Am Acad Dermatol 1996 34 804 14 10.1016/S0190-9622(96)90018-3 8632078

[B30] Hengge UR Ruzicka T Tyring SK Stuschke M Roggendorf M Schwartz RA Update on Kaposi's sarcoma and othe HHV8 associated diseases. Part 1: epidemiology, environmental predispositions, clinical manifestations, and therapy Lancet Infect Dis 2002 2 281 92 10.1016/S1473-3099(02)00263-3 12062994

[B31] Helm F Burgess G Helm F Kaposi's hemorrhagic sarcoma Cancer Dermatology 1979 Philadelphia: Lea &c Febiger 177 84

[B32] Kolios G Kaloterakis A Filiotou A Nakos A Hadziyannis S Gastroscopic findings in Mediterranean Kaposi's sarcoma (non-AIDS) Gastrointest Endosc 1995 42 336 9 10.1016/S0016-5107(95)70133-8 8536903

[B33] Cox FH Helwig EB Kaposi's sarcoma Cancer 1959 12 289 98 10.1002/1097-0142(195903/04)12:2<289::AID-CNCR2820120213>3.0.CO;2-Z 13638949

[B34] Iscovich J Boffetta P Brennan P Classic Kaposi's sarcoma as a first primary neoplasm Int J Cancer 1999 80 173 177 10.1002/(SICI)1097-0215(19990118)80:2<173::AID-IJC2>3.0.CO;2-2 9935195

[B35] Sapienza G Nasca MR Dinotta F Micali G Guess what. Classic Kaposi's sarcoma Eur J Dermatol 2001 11 157 8 11275818

[B36] Brenner B Weissmann-Brenner A Rakowsky E Weltfriend S Fenig E Friedman-Birnbaum R Classical Kaposi sarcoma: prognostic factor analysis of 248 patients Cancer 2002 95 1982 7 10.1002/cncr.10907 12404293

[B37] Krigel RL Laubenstein LJ Mucigia FM Kaposi's sarcoma: a new staging classification Cancer Treat Rep 1983 67 531 534 6861160

[B38] Mitsuyasu RT Groopman JE Biology and therapy of Kaposi's sarcoma Semin Oncol 1984 11 53 59 6538993

[B39] Giraldo F Beth F Huang ES Kaposi's sarcoma and its relationship to cytomegalovirus (GMV) III: GMV .1 DNA and early antigens in Kaposi's sarcoma./nZ/-.r' Cancer 1980 26 23 9 10.1002/ijc.2910260105 6263803

[B40] Hiatt Kim M Nelson Ann M Lichy Jack H Fanburg-Smith Julie C Classic Kaposi Sarcoma in the United States over the last two decades: Aclinicopathologic and molecular study of 438 non-HIV-related Kaposi Sarcoma patients with comparison to HIV-related Kaposi Sarcoma Modern Pathology 2008 21 572 582 10.1038/modpathol.2008.15 18376387

[B41] Caccialanza M Marca S Piccinno R Eulisse G Radiotherapy of classic and human immunodeficiency virus-related Kaposi's sarcoma: results in 1482 lesions JEADV 2008 22 297 302 18269597 10.1111/j.1468-3083.2007.02405.x

[B42] Bonnet F Morlat P Cancers et infection par le VIH: quelles associations ? La Revue de médecine interne 2006 27 227 235 10.1016/j.revmed.2005.10.003 16337065

[B43] Feurman EJ Portruch-Eisenkraft S Kaposi's sarcoma Dermatologica 1973 146 115 122 10.1159/000252034 4711877

[B44] Fenig E Brenner B Rakowsky E Classic Kaposi sarcoma: experience at Rabin Medical Center in Israel Am J Clin Oncol 1998 21 498 500 10.1097/00000421-199810000-00016 9781608

[B45] Digiovana JJ Safai B Kaposi's sarcoma: a retrospective study of 90 cases with particular emphasis on the familial occurence, ethnic background and prevalence of other diseases Am J Med 1981 71 779 782 10.1016/0002-9343(81)90364-8 7304649

[B46] Lothe F Kaposi's sarcoma in Ugandian Africans ACTA Pathol Microbiol Scand 1963 161 Suppl. 1 71 5877051

[B47] Perniciaro C Gross DJ White JW Familial Kaposi's sarcoma Cutis 1996 57 220 2222 8727769

[B48] Dupin N Maladie de Kaposi Rev M&d Interne 1995 16 484 486 10.1016/0248-8663(96)80743-0 7569416

